# Impact of three commonly used blood sampling techniques on the welfare of laboratory mice: Taking the animal’s perspective

**DOI:** 10.1371/journal.pone.0238895

**Published:** 2020-09-08

**Authors:** Neele Meyer, Mareike Kröger, Julia Thümmler, Lisa Tietze, Rupert Palme, Chadi Touma

**Affiliations:** 1 Department of Behavioural Biology, University of Osnabrück, Osnabrueck, Germany; 2 Research Group of Psychoneuroendocrinology, Max Planck Institute of Psychiatry, Munich, Germany; 3 Unit of Physiology, Pathophysiology and Experimental Endocrinology, University of Veterinary Medicine Vienna, Vienna, Austria; Technion Israel Institute of Technology, ISRAEL

## Abstract

Laboratory mice are the most frequently used animals in biomedical research. In accordance with guidelines for humane handling, several blood sampling techniques have been established. While the effects of these procedures on blood quality and histological alterations at the sampling site are well studied, their impact on the animals’ welfare has not been extensively investigated. Therefore, our study aimed to compare three commonly used blood sampling techniques regarding their effects on different indicators of animal welfare, including physiological and behavioural response stress parameters, including pain measures, home-cage behaviour and nest-building as well as exploratory activity and neophobia. Male C57BL/6J mice were subjected to a single blood collection from either the *vena facialis*, the retrobulbar sinus or the tail vessel, or were allocated to the respective control treatment. While all blood sampling techniques led to an acute increase in plasma corticosterone levels, the response was strongest in animals that underwent sampling from the *vena facialis* and the retrobulbar sinus. Similar results were observed when the time-course of adrenocortical activity was monitored via corticosterone metabolites from faecal samples. Blood collection from the *vena facialis* and the retrobulbar sinus also decreased exploration of novel stimuli, resulted in decreased nest-building activity and induced higher scores in the Mouse Grimace Scale. Moreover, locomotor activity and anxiety-related behaviour were strongly affected after facial vein bleeding. Interestingly, tail vessel bleeding only induced little alterations in the assessed physiological and behavioural parameters. Importantly, the observed effects in all treatment groups were no longer detectable after 24 hours, indicating only short-term impacts. Thus, by also taking the animal’s perspective and comprehensively assessing the severity of the particular sampling procedures, the results of our study contribute to *Refinement* within the 3R concept and allow researchers to objectively select the most appropriate and welfare-friendly blood sampling technique for a given experiment.

## Introduction

Laboratory mice are by far the most widely used vertebrate species in biomedical research [1]. In this research domain, it is often necessary to collect blood samples. Yet, blood sampling in mice potentially causes distress and could impact the health and welfare of the experimental animal and might bias the obtained results. Hence, for ethical and scientific reasons, the applied techniques for blood collection must cause as little pain and distress to the mouse as possible [2].

Accordingly, scientific organisations such as the *Society of Laboratory Animal Science* and the *National Centre for the Replacement*, *Refinement & Reduction of Animals in Research* published recommendations and guidelines for commonly used blood sampling techniques in laboratory mice [3, 4]. Amongst other methods, blood collection from the *vena facialis* (facial vein bleeding, FVB), the retrobulbar venous sinus (retrobulbar bleeding, RBB), and the tail vessels (tail vessel bleeding, TVB) are recommended for non-terminal blood collection [3–5]. However, these blood collection techniques differ in their degree of invasiveness, foremost because anaesthesia or restraint of the animal is necessary, and also because handling duration might vary considerably [6–9], which might result in different degrees of distress in the animals.

Russell and Burch proposed the “3Rs” *Replacement*, *Refinement* and *Reduction* in 1959 to minimise pain and distress for animals in biomedical research [10, 11]. To make a step towards *Refinement* concerning blood sampling in laboratory mice, our in-depth study comprehensively investigated the stress response caused by the three mentioned frequently used blood sampling techniques by also considering the animals’ perspective. Earlier studies have shown that the different sampling techniques affect the quality of the blood sample, change several biochemical parameters, and cause varying tissue damage at the sampling site [7, 12–18]. However, the assessment of animal welfare in response to stressful sampling procedures in an objective and scientifically reliable manner is challenging. Here, several parameters, especially regarding spontaneous and natural behaviours, present valuable information [19–23] but are frequently neglected in studies evaluating blood sampling techniques in laboratory mice.

There are already indications that the different blood sampling procedures may lead to alterations in spontaneous as well as experimentally induced behaviours and also physiological parameters; however, the results are ambiguous [17, 24–26]. These discrepancies are likely due to the fact that the cited studies differ in details of the applied technique as well as the frequency of blood sampling, and the time point after the procedure that the physiological and behavioural changes were assessed. Thus, sound evidence is still needed to make valid recommendations regarding appropriate and most welfare-friendly blood sampling techniques [27].

The present study, therefore, aimed to objectively and comprehensively compare the three commonly applied blood sampling techniques detailed above (TVB, RBB, FVB and the respective control treatments), with regards to their acute and medium-term (i.e. 24 hours post-treatment) effects on laboratory mice. To gather in-depth information about possible impacts on different welfare-related parameters, a variety of physiological as well as behavioural indicators were assessed in four separate experiments. These experiments were performed in male C57BL/6J mice, one of the most widely used inbred strain of mice in biomedical research and background strain to most genetically modified mouse models [28, 29], i.e. making the results relevant for a broad field.

Considering the seemingly different degree of invasiveness of the three blood sampling techniques, differences in stress hormone secretion, i.e. levels of plasma corticosterone and faecal corticosterone metabolites, were expected [30, 31]. Further indicators of decreased animal welfare would be altered spontaneous behaviours [32], as well as anxiety-related behaviours in familiar and novel environments [32–36], which we assessed via home-cage activity measurement and standardized behavioural tests, such as the Open Field, Novel Object Exploration and Social Interaction test. Moreover, nest-building and nest quality can be used as an indicator of animal welfare [20, 37–39], as laboratory mice are highly motivated to build nests when provided with appropriate nesting material [39]. To investigate whether the different blood sampling techniques resulted in changes in nest-building behaviour, we also assessed nest quality on several acute and medium-term time points following the sampling procedure. Recently, the facial expression of rodents has been used to assess welfare-related parameters [33, 34], as similar to humans, also mice show a so-called pain-grimace [40]. These changes in facial expression resulting from pain or distress can be quantified using the Mouse Grimace Scale (MGS), and increased scores are a strong indicator for compromised animal welfare [40]. Therefore, taking the animals perspective, we studied whether the mice showed alterations in the MGS score following the different blood sampling techniques. We hypothesised a differential impact of the blood sampling techniques on all these readouts, with more invasive techniques leading to the strongest impairment compared to control treatments.

## Methods

### Animals

**Fig 1:**
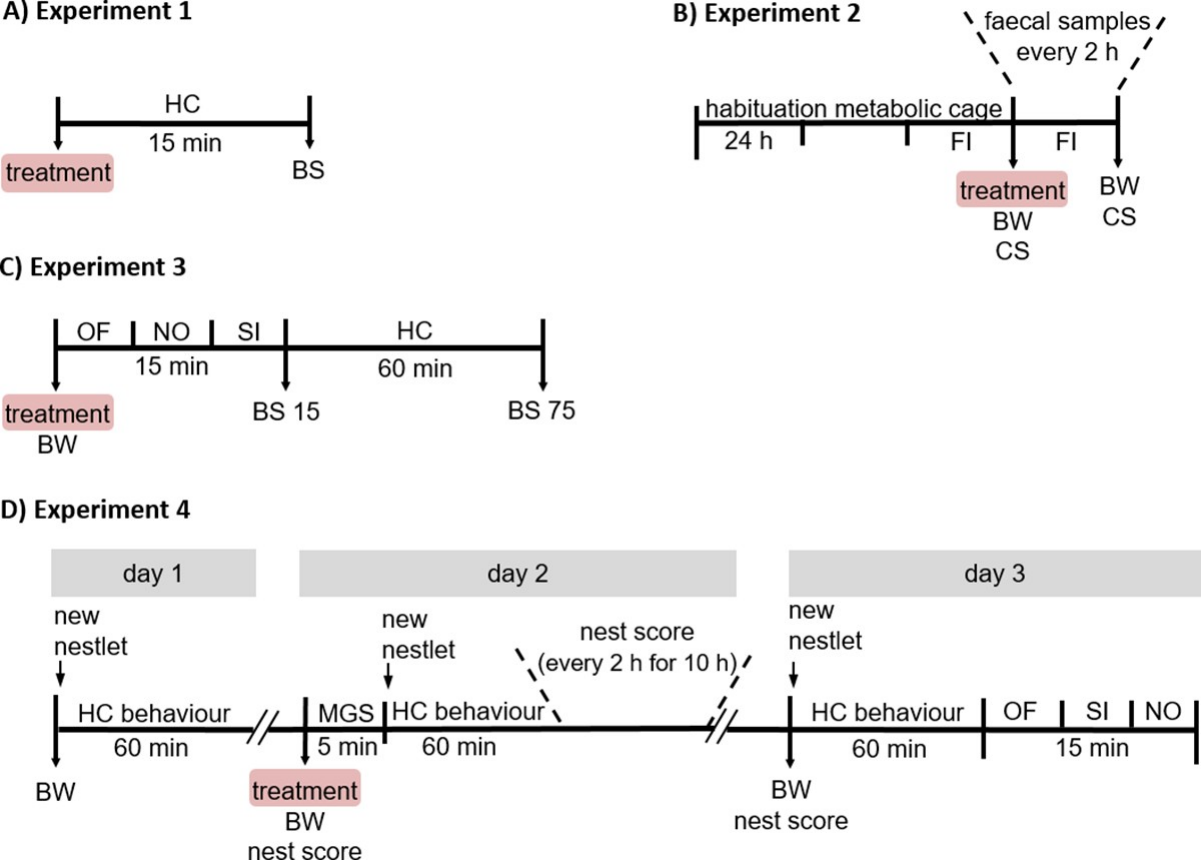
Schematic timeline of the four experiments. Male C57BL/6J mice were investigated in four different experiments (A–D). The treatment comprises one of the three blood sampling techniques (tail vessel bleeding (TVB), retrobulbar bleeding (RBB) or facial vein bleeding (FVB)) or one of the two control treatments (handling control (HCO) or anaesthesia control (ACO)). (A) In experiment 1, animals were subjected to the respective treatment and the acute stress hormone release was assessed 15 minutes later. (B) In experiment 2, faecal samples of the animals were collected every 2 hours for 24 hours following the treatment to assess the time course of HPA axis activation. In addition, food intake was measured and body weight as well as coat state were determined as indicators for animal welfare. (C) In experiment 3, the mice underwent three behavioural tests (5 minutes each) following the respective treatment to investigate acute effects on locomotor activity and anxiety-related behaviour. In addition, acute (BS 15) and recovery of (BS 75) stress hormone release was assessed. (D) In experiment 4, acute effects of the respective treatment on subjective pain perception (MGS), nest-building behaviour and home-cage behaviour was studied (day 2). In addition, medium-term effects on the nest-building and home-cage behaviour as well as locomotor activity and anxiety-related behaviour were assessed (day 3). Abbreviations: BS, blood sampling; BW, body weight; CS, coat state; FI, food intake; HC, home-cage; MGS, mouse grimace scale; NO, Novel Object test; OF, Open Field test; SI, Social Interaction test.

Young adult male C57BL/6J mice at the age of 10 to 12 weeks were subjected to one of three different blood sampling techniques or two control treatments in one of four experiments (schematic description in Fig 1). The animals were randomly allocated to the different treatment groups. After an *a priori* sample size calculation (G*Power, release 3.0.10) we aimed at including 12 animals per treatment and experiment, thus, in total 240 animals were tested. Deviations from this sample size are due to sample losses or other technical problems. Actual sample sizes are given in the corresponding figures and tables. The mice were obtained from a commercial breeder (Charles River, Sulzfeld, Germany) at the age of three weeks and were housed in a conventional housing system in open-topped Marcolon cages Type II long or Type III in groups of up to five animals. For experimental purposes and to avoid dominance hierarchy effects on behavioural and physiological read-outs, mice were individually housed in Macrolon cages Type II one week before the start of the respective experiment (experiment 1–4).

Cages were changed weekly, and food (Altromin No. 1324, Altromin GmbH, Lage, Germany) and water were provided *ad libitum*. Unless noted differently, cages were equipped with bedding material (LTE E-001, ABEDD Vertriebs GmbH, Vienna, Austria) and wood shavings (NBF E-011, ABEDD Vertriebs GmbH, Vienna, Austria) as nesting material and animals were handled using the tail-handling method. The housing rooms were kept at a 12:12 light-dark cycle (lights on at 8 am) with a constant room temperature of 22 ± 2°C and relative humidity of 55 ± 10%. The presented work complies with current regulations covering animal experimentation in Germany and the European Union (European Directive 2010/63/EU). All experiments were announced to and approved by the Lower Saxony State Office for Consumer Protection and Food Safety (LAVES, licence 19/3083) and the ‘Animal Welfare Officer’ of the University of Osnabrück.

### Blood sampling techniques

Three different blood sampling techniques were applied, comparing tail vessel bleeding (TVB), *vena facialis* bleeding (FVB) and the retrobulbar venous sinus bleeding (RBB). Furthermore, two control groups were included, i.e. handling control (HCO) and anaesthesia control (ACO). The blood sampling procedures and control treatments were identical for all four experiments and are described in detail below. In general, for all procedures, the mouse was transported in its home-cage to an adjacent experimental room, where the blood sampling or control treatments were performed. Immediately before any of the different treatments, the mice were weighed (Exp 2–4). The time from first disturbance of the home-cage until the mice were placed back in their cages did not exceed 3 minutes for all five treatment groups. Initial blood sampling was always performed in the first hours of the light phase, i.e. in the trough of the circadian corticosterone rhythm [41, 42]. Within an experiment, all blood sampling procedures were performed by the same trained and experienced experimenters.

#### Tail Vessel Bleeding (TVB)

The mouse was placed on a stainless steel grid (cage lid), and the tail was gently fixed between the fingers of the experimenter. Other than that, the mouse was allowed to move freely. With a scalpel, a small horizontal incision was made a few centimetres from the base of the tail nicking the ventral tail vessels [9, 43]. Blood was collected using EDTA-coated microvettes (CB 300, SARSTEDT AG & Co. KG, Nürmbrecht, Germany) up to a maximum volume of 150–250 μl. Gentle upward strokes from the base to the incision were applied when necessary to enhance blood flow. After collection, the bleeding was stopped by applying slight pressure on the incision using a cotton pad. Afterwards, the mouse was placed either back in its home-cage (Exp 1), in a metabolic cage (Exp 2) or a fresh holding cage (Exp 3 & 4). Blood samples were kept on ice until further processing.

#### Retrobulbar venous sinus bleeding (RBB)

The mouse was briefly anaesthetised in a glass chamber using a mixture of 5% isoflurane (Forene®, Abb.Vie GmbH& Co. KG, Germany) and ambient air. Once righting reflex was lost (usually after about 30 seconds), the mouse was removed from the anaesthesia chamber and was gently scruffed by the neck. A 10 μl capillary (Hirschmann Laborgräte GmbH & Co. KG, Eberstadt, Germany, outer diameter 0.8 mm) was inserted into the medial canthus of the right eye while applying gentle pressure and rotating the capillary carefully. No more than 250μl of blood was collected in an EDTA coated tube (1ml; Kabe Labortechnik GmbH, Nümbrecht-Elsenroth, Germany). Following blood collection, the capillary was gently removed, the eyelid was closed, and gentle pressure was applied with a cotton pad to minimise haemorrhage. The mice did not show any sign of consciousness during the sampling procedure. Afterwards, either the mouse was placed back in its home-cage (Exp 1), or in a metabolic cage (Exp 2), or a fresh holding cage (Exp 3 & 4), and the recovery from anaesthesia (usually within one minute) was monitored. Samples were kept on ice until further processing.

#### Vena Facialis Bleeding (FVB)

Blood sampling from the *vena facialis* was performed as described by Golde and colleagues [8]. Briefly, the mouse was scruffed by the neck and with a lancet (5.0 mm; Solofix®, B. Braun Melsungen AG, Melsungen, Germany) the *vena facialis* of the right cheek was punctured. The blood vessel is located approximately 3–4 mm dorsal-caudal to the hairless spot on the side of the jaw. Drops of blood were caught using EDTA-coated microvettes (CB 300, SARSTEDT AG & Co. KG; Nürmbrecht, Germany). Bleeding was stopped by applying gentle pressure with a cotton swab on the puncture site and afterwards, either the mouse was placed back in its home-cage (Exp 1), in a metabolic cage (Exp 2) or a fresh holding cage (Exp 3). No more than 150–250 μl of blood were collected, and samples were kept on ice until further processing.

#### Anaesthesia control (ACO)

To assess the impact of the inhalation anaesthesia applied for the RBB, an anaesthesia control group was included. The mice were treated identical to the RBB group, but without the blood sampling, i.e., the animal was placed in the anaesthesia chamber containing a mixture of 5% isoflurane (Forene®, Abb.Vie GmbH& Co. KG, Germany) and ambient air. Once the righting reflex was lost (usually after about 30 seconds), the mouse was removed from the chamber and placed either back in its home-cage (Exp 1), in a metabolic cage (Exp 2), or a fresh holding cage (Exp 3 & 4), and recovery from anaesthesia (usually within one minute) was monitored.

#### Handling control (HCO)

Since handling can also elicit a stress response, a handling control group was included. Here, mice were subjected to a short period of gentle handling of approximately 30 seconds, during which the mice were weighed. Afterwards, the mouse was placed either back in its home-cage (Exp 1), in a metabolic cage (Exp 2) or a fresh holding cage (Exp 3 & 4).

### Experiment 1—Effects on acute stress hormone release

In order to investigate the effects of the three different blood sampling techniques and the two control procedures on acute HPA axis activation, the mice were subjected to one of the five treatments described above. After returning the mice to their home-cages for 15 minutes, a second blood sample was collected for the measurement of plasma corticosterone concentrations. For this, the animals were quickly sacrificed by decapitation under isoflurane anaesthesia and trunk blood was collected into EDTA coated tubes (Kabe Labortechnik GmbH, Nümbrecht-Elsenroth, Germany) containing 10 μl of a protease inhibitor solution (1.54 μM aprotinin, Carl Roth GmbH & Co.KG, Karlsruhe, Germany). Samples were kept on ice until further processing.

### Experiment 2 –Effects on the time course of HPA axis activation and welfare-relevant behavioural indicators

In order to assess effects of the three blood sampling techniques on the time course of HPA axis activation, the animals were housed in so-called metabolic cages (stainless steel wire cages type III, 38 x 22 x 15 cm) equipped with a paper towel as nesting material to aid thermoregulation of the animals [for details see 44, 45]. The mice were transferred to these cages already three days before the start of the experiment in order to habituate them to the new housing condition and the faecal sampling procedure [44, 45]. After habituation, mice were subjected to one of the five treatments detailed above. During the next 24 hours, faecal samples were collected every two hours and stored -20°C until further processing [43, for details see 44, 45].

To assess effects of the different treatments on food intake, the amount of food consumed in 24 hours was measured for each mouse on the day before the respective treatment and the day afterwards. The animal’s coat state was assessed right before the treatment and 24 hours later. The coat state was scored following previously published protocols [for details see 46, 47]. Briefly, the coat state represented the sum of scores (0 = well-groomed, 1 = unkempt) obtained from seven different body parts: head, neck, dorsal coat, ventral coat, base of the tail, front legs, and hind legs. After an observation period of one week, animals were euthanized by isoflurane anaesthesia followed by rapid decapitation.

### Experiment 3—Effects on acute stress hormone release and recovery, and impact on locomotor activity, exploration and anxiety-related behaviour

In order to further investigate the effects of the different blood sampling techniques, the body weight was assessed on the day before (-24 h), immediately before (0 h) and one day after the respective treatment (24 h). Locomotor activity and explorative behaviour of non-social and social stimuli, a series of three tests was conducted directly following the respective treatment. The Open Field (OF), Novel Object (NO) and Social Interaction (SI) test were performed in direct succession, each lasting five minutes. Hereby, each test served as a habituation for the next test, i.e. the OF test habituated the animal to the arena in which a novel object (wire pencil holder) was placed during the NO test. The NO test served to habituate the animal to the wire pencil holder under which the unknown male conspecific was placed during the SI test. To study acute stress hormone release and recovery, a blood sample was taken directly after the SI test (t = 15 min) again 60 minutes later (t = 75 min) by TVB. Between these two blood samplings, the animals were left undisturbed and stayed in their home-cages in the housing room.

#### Behavioural tests

The OF, NO, and SI were performed directly after each other in the same round test arena (Ø 60 cm, made of black PVC), dimly lit (approximately 15 lux) and lasted for five minutes each. At the beginning of the OF test, the mice were placed in the outer zone of the arena facing the wall and were allowed to explore the new environment for five minutes. Afterwards, the animals were briefly removed from the arena and a novel object (wire pencil holder, 10 x 10 x 11 cm (L x W x H) DOKUMENT; Ikea, Germany, S1 Fig) was placed in the middle of the arena for the NO test. The animal was returned to the arena and was allowed to explore the now known OF arena and the novel object for five minutes. Afterwards, the mouse was again briefly removed from the arena. For the SI test, an unfamiliar male mouse of the CD-1 strain (Crl:CD-1(ICR)) was placed underneath the wire pencil holder as a social interaction partner. This allowed for olfactory, visual and auditory but not physical contact between the test animal and the interaction partner. Then the mouse could freely explore the arena and the social partner for five minutes. All three behavioural tests were video-recorded using a digital video camera, and the arena was divided in an outer and inner zone (30 cm Ø). Additionally, in the NO and SI, an interaction zone was defined at a distance of 2.5 cm around the object/social partner. The total distance travelled, the distance travelled in, the number of entries to and the time spent in each zone was automatically recorded using the tracking software *ANY-maze* (Stoelting Europe, Ireland, version 4.99). An entry was counted when at least 80% of the body of the mouse was present in the particular zone (inner and outer zone), or the head entered the interaction zone. After an observation period of one week, animals were euthanized by isoflurane anaesthesia followed by rapid decapitation.

### Experiment 4 –Effects on spontaneous behaviour, pain perception, and medium-term effects on locomotor activity, exploration and anxiety-related behaviour

In order to assess spontaneous behaviour in response to the different treatments, we investigated home-cage behaviour and nest-building activity. Moreover, to assess pain perception of the mice, their facial expression was studied using the Mouse Grimace Scale (MGS). In the week prior to this experiment, the mice were provided two times with nesting material (Nestlets) made from pressed cotton fibres (Ancare Corp., Bellmore, New York, USA), in order to familiarise them with this new nesting material. Moreover, to habituate the test animals to the MGS recording procedure, they were placed in the MGS *boxes* (details see below) on three consecutive days for five minutes each. The boxes were made from acrylic glass with a hinged-lid (10 x 10 x 5 cm (L x W x H), S2 Fig).

#### Home-cage behaviour

Home-cage behaviour was studied to examine whether the different blood sampling methods or control treatments led to differences in natural and unprovoked behaviour patterns. For this purpose, mice were recorded using a webcam (Full-HD-webcam Besteker 1536P) in their home-cages (lateral view on the cage) while positioned at the usual place in the housing rack. Home-cage behaviour was assessed for 60 minutes each one day prior, immediately following the respective treatments and 24 hours afterwards. The behavioural analysis was performed by an observer blind to the treatment of the animals using the BORIS software (version 6.2.4) [48]. Descriptions of the scored behaviours are listed in Table 1.

**Table 1:** Descriptions of the behaviours scored in the home-cage.

Behaviour	Definition
*Maintenance behaviour*	
Feeding	The mouse rears up to gnaw at food pellets through the bars of the food hopper. Alternatively, the mouse uses its forepaws to hold the food pellet while gnawing.
Drinking	The mouse rears up and licks the nozzle of the water bottle.
Grooming	The mouse licks its fur or moves its front paws over the body through the fur. Alternatively, scratching motions with any limb.
Nest-building	The mouse manipulates the nesting material (Nestlet) using its snout or paws.
*Inactivity*	
Resting	The mouse is lying curled up on its side or is sitting curled up. The head may be tucked under the body.
Immobile with a hunched posture	The mouse is sitting and motionless with a strong curvature of the back. The ears are pulled back and the limbs are pulled in close under the body
*Exploration*	
Locomotion	The mouse moves its body forward by walking or running.
Digging	The mouse is moving bedding material by shovelling or pushing it in a forward motion. Mostly forepaws are used; hind paws and head may assist.
Rearing	The forepaws are lifted from the ground and the head is lifted. The back is straight. One or both forepaws may be placed at the cage wall.
*Miscellaneous*	
Time out in the nest	The mouse is in the nest and it is not detectable what it is doing.
Undefined	The mouse is outside the nest, but the behaviour is not clearly visible or not defined in the list of quantified behaviours.

#### Nest-building behaviour

Nest-building is a natural behaviour exhibited by small rodents and was shown to be an indicator for the welfare of laboratory mice [49]. We assessed nest-building behaviour using the nest building scores described by Deacon [37]. Briefly, a score between 1 and 5 was assigned depending on how much of the provided Nestlet was shredded and how much of it was used for nest-building. A score of 1 means that more of 90% of the Nestlet is still intact, while a score of 5 is assigned when more than 90% of the Nestlet is torn, and a nest with walls was built (walls higher than body of the animals for more than 50% of the nest circumference) [37]. Nest-building was assessed on the morning right before the different treatments (baseline value). After the treatment, the old nesting material was removed and a fresh Nestlet was provided in the home-cage of the animals. Over the time course of 10 hours, nest scores were noted every two hours without disturbing the animals. The final nest score was assigned 24 hours after the respective treatment. Scoring was always performed by the same trained observer who was blind to the treatment.

#### Mouse Grimace Scale (MGS)

To assess whether pain perception in the experimental animals differed following the different treatments, their facial expression was investigated utilising the MGS. The MGS was first described by Langford and co-workers [40] as a measure of subjective pain in mice. Briefly, five facial features (facial action units) were scored as potential indices of pain and three levels of intensity were assigned (0 = baseline, 1 = moderate, 2 = severe). The sum of these scores constitutes the MGS score. These features included tightening of the orbital region, bulging of the nose, bulging of the cheek, ear position and changes in whisker position [40]. To investigate acute effects of the different treatments, we video-recorded the mice for 5 minutes in ‘MGS-boxes’ described above immediately after the respective treatment. From these recordings, screenshots were grabbed whenever the mouse faced the camera (Full-HD-webcam Besteker 1536P). From these images, six pictures per mouse were randomly chosen for scoring. Two independent, trained observers who were blind to the treatment of the animals performed the scoring, and their scores were averaged to determine the final MGS score. It should be noted that it was not possible to score the action unit ‘whisker position’ reliably and therefore, it was excluded from the analysis. Hence, the maximum MGS score the animals could reach was eight.

#### Behavioural tests 24 hours after the treatment

In order to investigate possible persisting (medium-term) effects of the different treatments on locomotor activity and exploration of novel objects and social partners the mice in experiment 4 underwent the same set of behavioural tests (OF, NO, SI) as described in experiment 3. These tests were performed directly after the one-hour recording of home-cage behaviour on the day following the treatment. After an observation period of one week, animals were euthanized by isoflurane anaesthesia followed by rapid decapitation.

### Endocrine analyses

#### Plasma corticosterone

Plasma corticosterone levels of blood samples collected in experiment 1 and 3 were analysed as described in detail elsewhere [43]. Briefly, blood samples were centrifuged for 10 minutes at 4°C at 4000 g, and plasma samples were analysed either using a commercial corticosterone radioimmunoassay (Rat/Mouse CORT ^125^I RIA Kit, DRG Instruments GmbH, Marburg, Germany) or a corticosterone ELISA kit (EIA 4164, DRG Instruments GmbH, Marburg, Germany). All samples were processed according to the manufacturer’s instructions with slight modifications detailed in [43]. All standards, samples, and controls were run in duplicate. Intra- and inter-assay coefficients of variation were below 10 and 12%, respectively.

#### Faecal Corticosterone Metabolites (FCM)

The collected faecal samples were analysed for immunoreactive CM using a 5α-pregnane-3β,11β,21-triol-20-one EIA. Details regarding development, biochemical characteristics and physiological validation of this assay are described by Touma and colleagues [44, 45]. Moreover, the utilized EIA has proven well suited to detect even small changes in adrenocortical activity in mice [31, 45]. Before EIA analysis, faecal samples were dried at 80°C for two hours. Afterwards, they were homogenised and aliquots of 0.05 g were extracted with 1 ml of 80% methanol. A detailed description of the assay performance has been published elsewhere [44].

### Statistical analysis

For group comparisons of the five treatments, ANOVA was performed (with Welch’s correction in case of unequal variance), followed by Bonferroni *post hoc* testing. Repeated-measures ANOVA was used to analyse the time course of FCM excretion. To meet the assumption of parametric analysis, residuals were examined using the Lilliefors corrected Kolmogorov-Smirnov test. In case data could not be transformed to reach normal distribution (e.g. nest test and home-cage behaviour) data were analysed non-parametrically, i.e. applying the Kruskal—Wallis H test to detect significant differences between the different treatment groups. In case of significant differences, *post hoc* Bonferroni corrected Mann-Whitney U tests were carried out. The Friedman test was used as a non-parametric test for repeated measures. All data were analysed using the software IBM SPSS Statistics (Version 25.0). Graphs were created with the software GraphPad Prism (Version 7.01). Data are presented as means + SEM. For all tests differences were considered significant if p < 0.05.

## Results

In order to comprehensively assess the severity of the different blood sampling techniques, we investigated the impact of the three blood sampling techniques on direct and indirect indicators of animal welfare in four separate experiments: the acute stress response (experiment 1) and the course of the stress response (experiment 2). Additionally, we assessed the acute and medium-term effects on locomotor activity and the exploration of novel objects and a social partner in a behavioural test situation (experiment 3 and 4). Moreover, acute and medium-term alterations in home-cage behaviour, nest-building, and subjective pain perception were examined (experiment 4). In addition to the three blood sampling groups (tail vessel bleeding (TVB), retrobulbar bleeding (RBB) and facial vein bleeding (FVB)) we included two relevant control groups (handling control (HCO) and anaesthesia control (ACO)) in each experiment (details in Methods section).

### The three blood sampling techniques differentially affected acute stress hormone release

**Fig 2:**
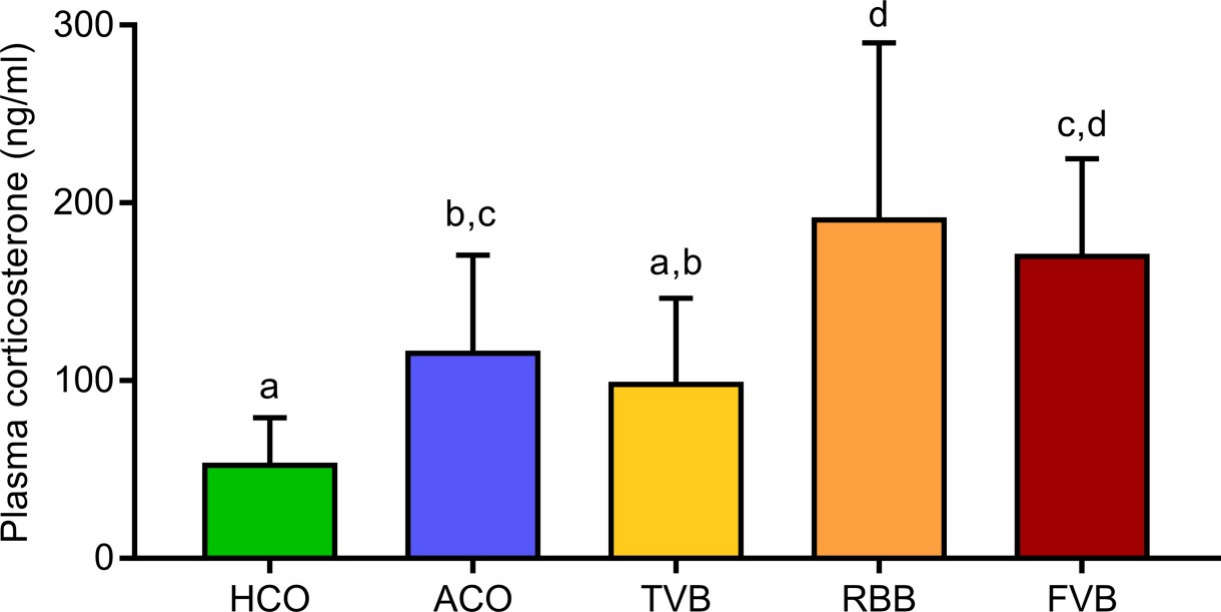
Plasma corticosterone levels 15 minutes after blood sampling or control treatment. Statistics: ANOVA; *post hoc* testing: Bonferroni, n = 12 per treatment group. Significant differences between groups are indicated by different letters. Data are presented as means and SEM. The underlying numerical data of the figure are available in the supporting information (S1 Data). HCO, handling control; ACO, anaesthesia control; TVB, tail vessel bleeding; RBB, retrobulbar bleeding; FVB, facial vein bleeding.

In the first experiment (see schematic description in Fig 1A), we investigated the acute stress hormone response, i.e. whether the treatment groups differed significantly in their plasma corticosterone levels 15 minutes after the respective treatment. Indeed, we detected a main effect of treatment (F_(4,55)_ = 13.727, p < 0.001, Fig 2). Compared to the HCO group, plasma corticosterone levels were significantly increased in animals from the ACO (p = 0.011), RBB (p < 0.001) and FVB (p < 0.001) groups. Interestingly, animals from the TVB group did not differ significantly from the HCO group (p > 0.05). Moreover, mice that only underwent isoflurane anaesthesia (ACO) showed a significantly lower plasma corticosterone response than animals from the RBB group (p = 0.036). Additionally, plasma corticosterone levels were significantly lower in animals that underwent TVB compared to RBB (p = 0.003) and FVB (p = 0.018). Mice from the RBB and FVB groups showed highest levels of plasma corticosterone and did not differ significantly from each other (p > 0.05).

### The three blood sampling techniques induced different degrees of HPA axis activation over several hours

**Fig 3:**
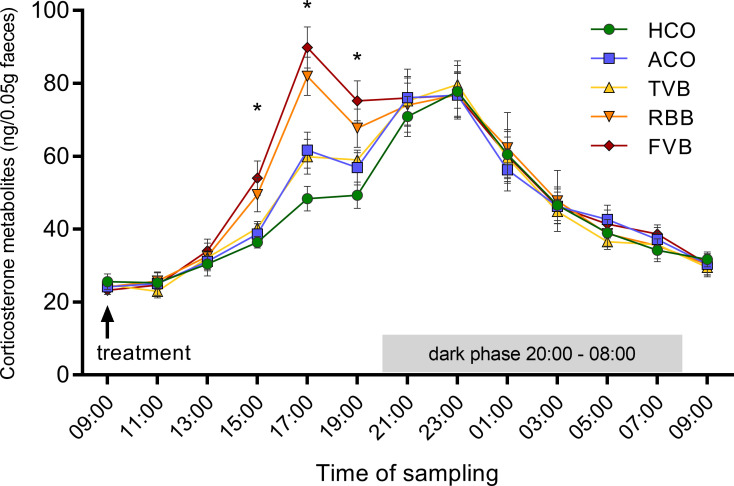
Faecal corticosterone metabolites monitored over 24 hours following blood sampling or control treatment. Faecal samples were collected in two-hour intervals. Data are presented as means ± SEM. Statistics: ANOVA; *post hoc* testing: Bonferroni, n = 12 per treatment group. Main effect of treatment: * p < 0.05. Six hours after the respective treatment, animals that underwent FVB showed significantly higher values compared to HCO (p = 0.006) and ACO (p = 0.023). Eight hours after the respective treatment, RBB and FVB differed significantly from HCO (both: p < 0.001), ACO (p = 0.049 and p = 0.002, respectively) and TVB (p = 0.022 and p = 0.001, respectively). Ten hours after the respective treatment, RBB and FVB differed significantly from HCO (p = 0.049 and p = 0.001, respectively). The underlying numerical data of the figure are available in the supporting information (S1 Data). HCO, handling control; ACO, anaesthesia control; TVB, tail vessel bleeding; RBB, retrobulbar bleeding; FVB, facial vein bleeding.

In the second experiment (see schematic description in Fig 1B), the time course of the induced HPA axis stress response was investigated by monitoring faecal corticosterone metabolites (FCMs) over 24 hours following the respective treatments. Faecal samples were collected in two-hour intervals. All groups showed the regular diurnal variation of HPA axis activity in their FCM levels. However, a repeated-measures ANOVA detected a significant time*treatment effect (F_(20.12, 276.64)_ = 2.517, p < 0.001, Greenhouse-Geisser corrected, Fig 3). With the expected time delay for faecal samples [44, 45], a main effect of treatment was detected six hours (F_(4,55)_ = 4.981, p = 0.002), eight hours (F_(4,55)_ = 12.200, p < 0.001) and ten hours (F_(4,55)_ = 5.116, p = 0.001) after blood sampling or control treatment (Fig 3). *Post hoc* analyses revealed that 6 hours after FVB, FCM concentrations were significantly higher than in the HCO (p = 0.006) and ACO (p = 0.023) groups. Eight hours after the respective treatment, mice of the RBB and FVB groups showed significantly higher FCM concentrations compared to the HCO (RBB: p < 0.001, FVB: p < 0.001), ACO (RBB: p = 0.049, FVB: p = 0.002), and TVB groups (RBB: p = 0.022, FVB: p = 0.001). Ten hours after the respective treatments, FCM concentrations were still significantly higher in mice that underwent RBB and FVB compared to HCO animals (RBB: p = 0.049, FVB: p = 0.001).

### The three blood sampling techniques differentially affected locomotion, exploration and anxiety-related behaviours as well as acute and recovery plasma corticosterone levels

In the third experiment (see schematic description in Fig 1C), the mice underwent three different behavioural tests immediately following the respective treatment. In direct succession, the animals performed the Open Field test (OF), the Novel Object test (NO), and the Social Interaction test (SI), each lasting 5 minutes. These behavioural tests were followed by two plasma corticosterone measurements, immediately after the behavioural tests (t = 15, acute response value) and 60 minutes later (t = 75, recovery value). These two blood samplings were performed using the TVB technique in all five treatment groups.

**Fig 4:**
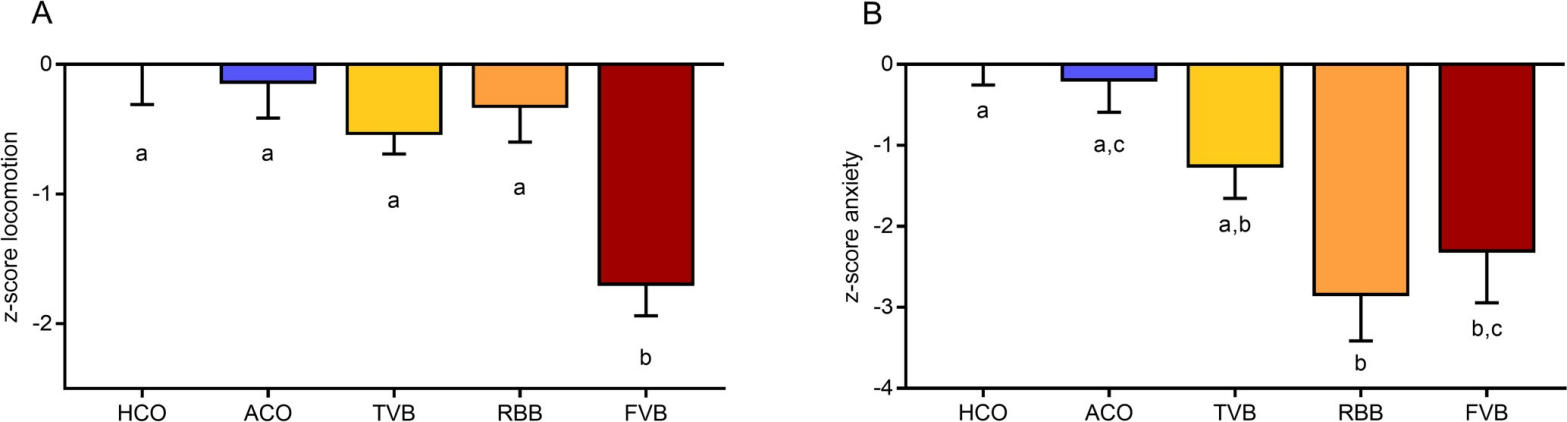
Locomotor activity and anxiety-related behaviour immediately after blood sampling or control treatment. *Z-scores* were calculated for locomotion (A) and anxiety (B) in the Open Field, Novel Object and Social Interaction test. Statistics: ANOVA with Welch’s test in case of unequal variances; *post hoc* testing: Bonferroni or Games-Howell in case of unequal variances. Significant differences between groups are indicated by different letters. n (HCO) = 10, n (ACO) = 10, n (TVB) = 9, n (RBB) = 11, n (FVB) = 11. Data are presented as means and SEM. The underlying numerical data of each figure are available in the supporting information (S1 Data). HCO, handling control; ACO, anaesthesia control; TVB, tail vessel bleeding; RBB, retrobulbar bleeding; FVB, facial vein bleeding.

For the analysis of the behavioural data, *z-scores* were calculated integrating measures along the same behavioural dimension [50]. We integrated ‘distance travelled’ for all three behavioural tests as a measure for locomotor activity (z-score locomotion, Fig 4A). Moreover, a measure for ‘anxiety-related behaviours’ was used, comprising the latency to enter, the time spent in, and the number of entries to the interaction zone and the time spent in the outer zone in the NO and SI tests (z-score anxiety, Fig 4B). A main effect of treatment was detected for both z-scores (z-score locomotion: F_(4,46)_ = 6.516, p < 0.001 and z-score anxiety: Welch’s F_(4,22.33)_ = 6.730, p = 0.001). While locomotion was generally reduced following the respective treatments compared to the HCO group, the FVB group showed significantly reduced locomotor activity compared to all other treatment and control groups (FVB vs HCO: p = 0.001, FVB vs ACO: p = 0.002, FVB vs TVB: p = 0.046, FVB vs RBB: p = 0.006; Fig 4A).

Regarding the z-score for anxiety-related behaviours (Fig 4B), following RBB and FVB the animals showed significantly higher levels of anxiety-related behaviours compared to HCO animals (Games-Howell *post hoc*: RBB vs HCO: p = 0.005, FVB vs HCO: p = 0.044). Moreover, RBB treated mice showed significantly increased anxiety-related behaviours compared to the ACO group (Games-Howell *post hoc*: p = 0.014). These effects were not detectable anymore 24 hours after the different treatments (tested in a different batch of animals, see Exp 4, z-scores locomotion: F_(4,44)_ = 0.719, p = 0.343, z-scores anxiety: F_(4,44)_ = 1.129, p = 0.355). Data and statistical analysis of the various parameters quantified in the three behavioural tests for both, immediate and medium-term effects can be found in S1 and S2 Tables.

**Fig 5:**
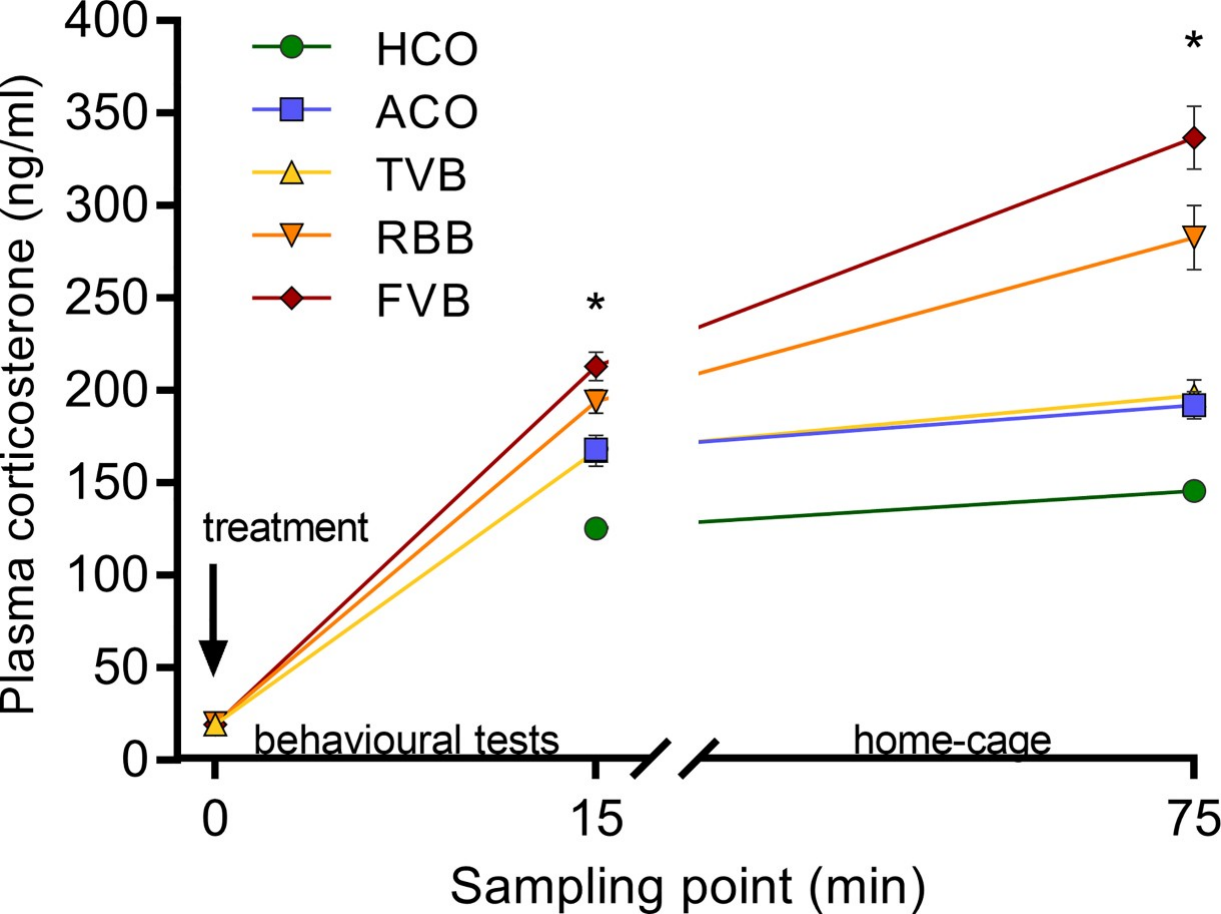
Baseline (0 min), acute (15 min) and recovery (75 min) plasma corticosterone levels after the respective treatment. Baseline values did not differ significantly between treatment groups. A significant difference (* p < 0.05) was detected between the treatment groups for the acute and recovery corticosterone levels. Acute levels were significantly lower in HCO animals compared to all other groups (ACO: p = 0.001, TVB: p = 0.001, RBB: p < 0.001, FVB: p < 0.001). In addition, animals that underwent FVB differed significantly from the ACO (p = 0.002) and TVB groups (p = 0.003). Recovery values were significantly lower in the HCO group compared to all other groups (p = 0.002, TVB: p = 0.002, RBB: p < 0.001, FVB: p < 0.001). Moreover, ACO and TVB groups differed significantly from RBB or FVB groups (Games-Howell *post hoc*: RBB vs ACO: p = 0.007, RBB vs TVB: p = 0.010, FVB vs ACO: p < 0.001, FVB vs TVB: p = 0.001). Statistics: ANOVA with Welch’s test in case of unequal variances; post hoc testing: Bonferroni or Games-Howell in case of unequal variances; at sampling point ‘0’ n (TVB) = 9, n (RBB) = 10, n (FVB) = 10; at sampling point ‘15’ n (HCO) = 10, n (ACO) = 10, n (TVB) = 9, n (RBB) = 12, n (FVB) = 11; at sampling point ‘75’ n (HCO) = 10, n (ACO) = 10, n (TVB) = 9, n (RBB) = 11, n (FVB) = 10. Data are presented as means ± SEM. The underlying numerical data of the figure are available in the supporting information (S1 Data). HCO, handling control; ACO, anaesthesia control; TVB, tail vessel bleeding; RBB, retrobulbar bleeding; FVB, facial vein bleeding.

Regarding the plasma corticosterone measurements, for both, the acute response value and the recovery value a significant main effect of treatment was detected (F_(4,46)_ = 20.549, p < 0.001 and Welch’s F_(4, 21.81)_ = 27.688, p < 0.001; Fig 5). The acute plasma corticosterone response was lowest in HCO animals compared to all other groups (Bonferroni *post hoc*: ACO: p = 0.001, TVB: p = 0.001, RBB: p < 0.001, FVB: p < 0.001; Fig 5). In addition to showing significantly higher corticosterone levels than the HCO animals, mice that underwent FVB also showed a significantly stronger increase in plasma corticosterone levels compared to ACO (p = 0.002) and TVB (p = 0.003) animals. Recovery values were again significantly lower in the HCO group compared to all other groups (Games-Howell *post hoc* ACO: p = 0.002, TVB: p = 0.002, RBB: p < 0.001, FVB: p < 0.001; Fig 5). Moreover, in comparison to the ACO and TVB groups, animals that underwent RBB or FVB showed significantly higher corticosterone levels (Games-Howell *post hoc*: RBB vs ACO: p = 0.007, RBB vs TVB: p = 0.010, FVB vs ACO: p < 0.001, FVB vs TVB: p = 0.001; Fig 5).

### The three blood sampling techniques acutely affected nest-building, pain perception and home-cage behaviour

In the fourth experiment (see schematic description in Fig 1D), we assessed the nest-building behaviour of the differently treated mice using the nest test protocol described by Deacon [37]. Moreover, we applied the Mouse Grimace Scale (MGS) [40] to assess pain-related behaviours and investigated changes in the home-cage behaviour of the animals.

**Fig 6:**
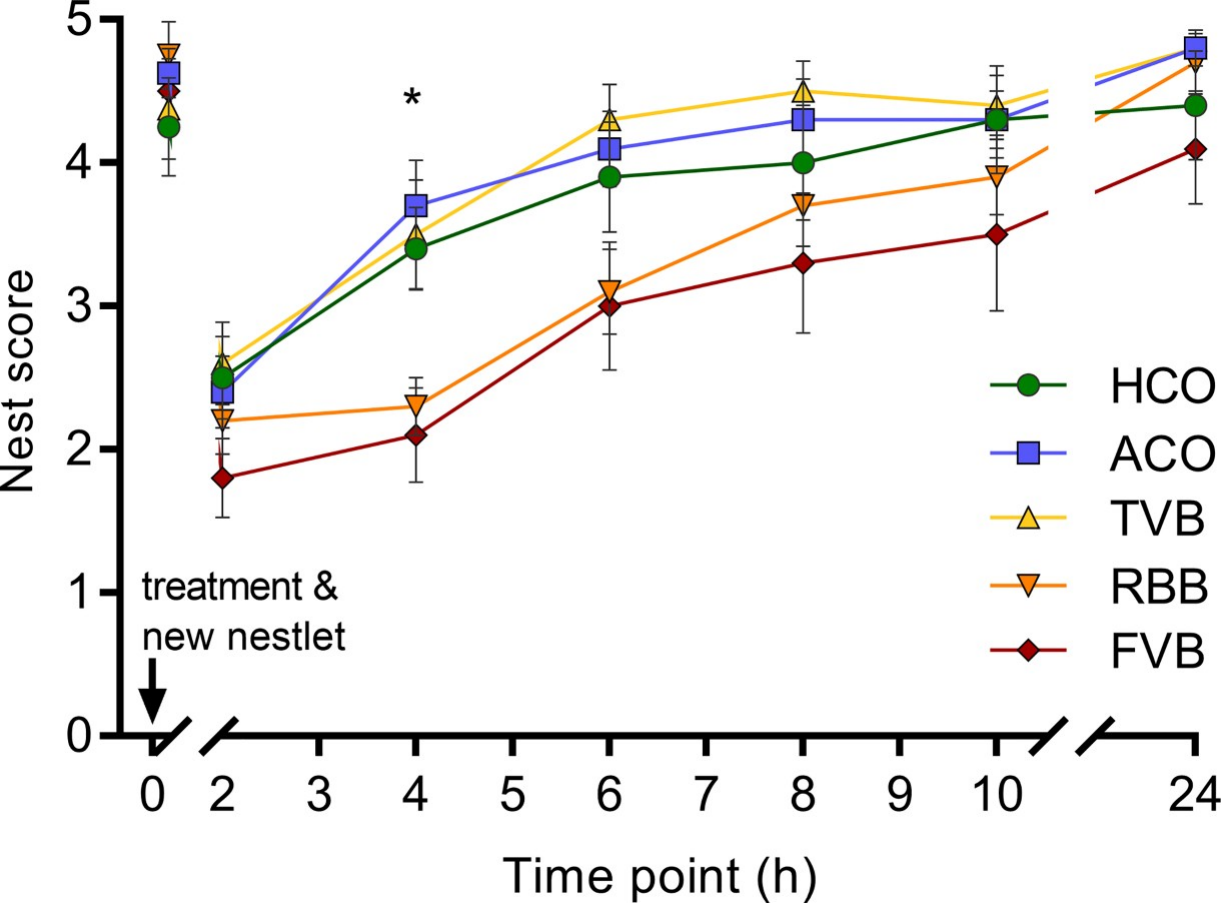
Effects of blood sampling or control treatment on nest-building behaviour. Nest quality was scored before (time point 0) and after the respective treatment. Immediately following the treatments, a new Nestlet was provided and nest quality was scored every 2 hours for 10 hours and again after 24 hours. Nest quality could reach a score between 1 (poor nest quality) to 5 (excellent nest quality) [37]. A significant difference (* p < 0.05) in nest quality between the groups was detected 4 hours after the respective treatment. Animals that underwent RBB and FVB differed significantly from animals of the ACO group (RBB: p = 0.023; FVB: p = 0.038). Statistics: Kruskal-Wallis H test followed by *post hoc* (Bonferroni-corrected Mann-Whitney U test), at time point ‘0’: n = 8 per group; at all other time points n (HCO) = 10, n (ACO) = 10, n (TVB) = 10, n (RBB) = 10, n (FVB) = 9. Data are presented as mean ± SEM. The underlying numerical data of the figure are available in the supporting information (S1 Data). HCO, handling control; ACO, anaesthesia control; TVB, tail vessel bleeding; RBB, retrobulbar bleeding; FVB, facial vein bleeding.

Nest quality was assessed immediately before the respective treatment (baseline value), over a 10-hour time course (in two hour intervals) immediately following the treatments (acute effects) and again 24 hours after the treatments (medium-term effects). Baseline nest scores did not differ between the five different treatment groups (Kruskal-Wallis H test: χ^2^ = 1.947, df = 4, p = 0.763; Fig 6). Over the ten hours immediately after the experimental treatments, a significant increase in nest scores across time was observed in all groups (Friedman test: χ^2^ = 30.715–38.321, df = 6, p < 0.001; Fig 6). A between treatment comparison at the different sampling points revealed a significant difference four hours after the respective treatment (χ^2^ = 16.422, df = 4, p = 0.003). Animals from the RBB and FVB groups achieved significantly lower nest-building scores compared to mice that underwent ACO (RBB: p = 0.023, FVB: p = 0.038, Bonferroni corrected Mann-Whitney U test). Yet, after 24 hours, no significant differences in nest scores were recorded between the treatment groups (Kruskal-Wallis H test: χ^2^ = 2.269, df = 4, p = 0.714), and most mice achieved a score of either 4 or 5, similar to baseline values (Fig 6).

**Fig 7:**
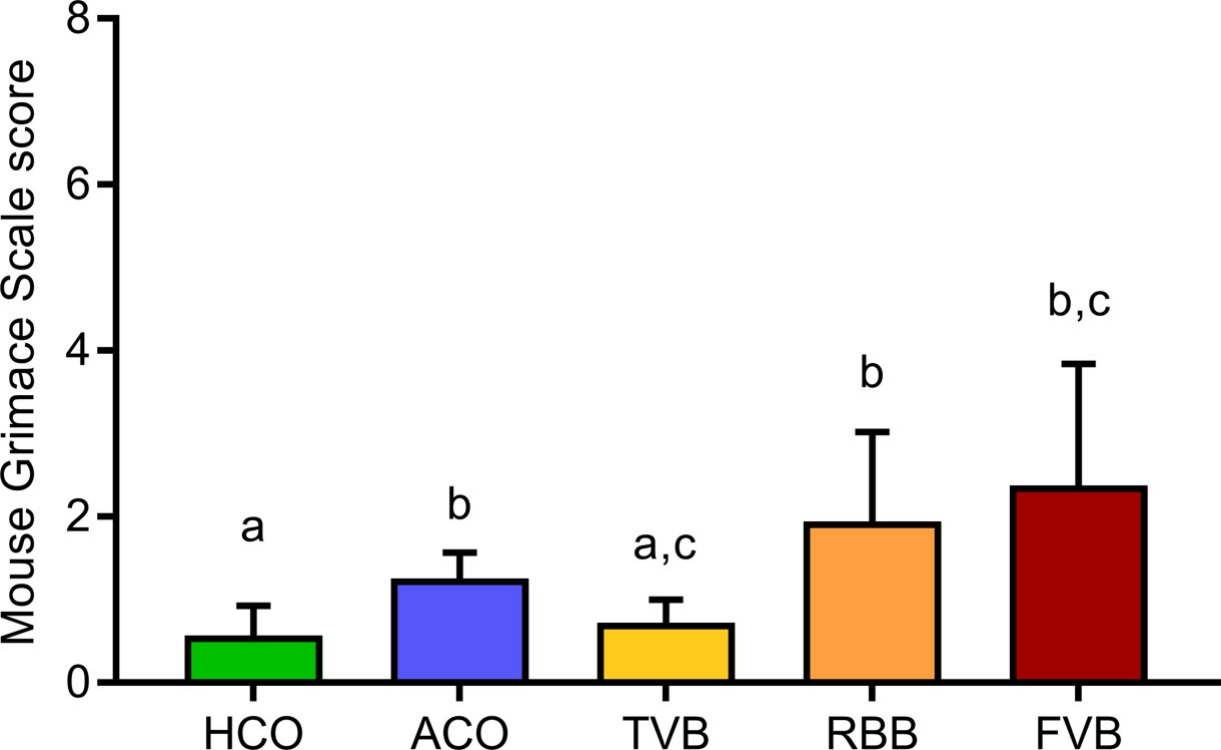
Effects of blood sampling or control treatment on pain perception as assessed in the Mouse Grimace Scale score (MGS). Four different facial features (orbital tightening, nose bulge, cheek bulge and ear position) were scored according to changes from baseline (0 = no change, 1 = moderate, 2 = severe) [40]; thus the maximum score that could be reached was 8. Statistics: ANOVA with Welch’s test; *post hoc* testing: Games-Howell. Significant differences between groups are indicated by different letters. n (HCO) = 10, n (ACO) = 9, n (TVB) = 9, n (RBB) = 10, n (FVB) = 9. Data are presented as means and SEM. The underlying numerical data of the figure are available in the supporting information (S1 Data). HCO, handling control; ACO, anaesthesia control; TVB, tail vessel bleeding; RBB, retrobulbar bleeding; FVB, facial vein bleeding.

In order to evaluate the subjective pain perception of the animals, their facial expression was scored in the 5 minutes immediately following the respective treatments, utilising the MGS scoring protocol [40]. Here, a main effect of treatment was detected (Welch’s F_(4,20.41)_ = 9.127, p < 0.001; Fig 7). *Post hoc* testing revealed significantly higher MGS scores for mice that underwent ACO, FVB or RBB compared to the HCO group (Games-Howell *post hoc*: p = 0.004, p = 0.030 and p = 0.015, respectively). While animals that underwent TVB did not differ significantly from the HCO and FVB groups (Games-Howell: p = 0.813 and p = 0.056), a significant difference in MGS score was detected compared to the ACO (p = 0.014) and RBB (p = 0.036) groups. The ACO, RBB and FVB groups did not differ significantly from each other (ACO vs RBB p = 0.466, ACO vs FVB p = 0.338, RBB vs FVB p = 0.973).

To assess the impact of blood sampling on the animals’ natural behaviour in the home-cage, we analysed their activity for 60 minutes at three time points: 24 hours before, directly after and 24 hours after the respective treatment. On the day before the blood sampling and control treatments, the animals did not differ significantly in their home-cage behaviour (see S3 Table). On the day of blood sampling, however, significant differences were detected between the treatment groups for several behaviours (see Table 2): The time spent grooming was significantly different between the treatment and control groups (χ^2^ = 11.598, df = 4, p = 0.021). *Post hoc* testing revealed that mice that underwent FVB showed significantly more grooming compared to mice from the HCO group. In addition, the treatment and control groups differed significantly regarding the number of rearings (χ^2^ = 14.61, df = 4, p = 0.006). Animals from the FVB group showed significantly fewer rearings compared to ACO mice (p = 0.018). Moreover, we observed a significant difference in the time the animals spent immobile while expressing a hunched body posture (χ^2^ = 26.058, df = 4, p < 0.001). This behaviour was almost exclusively shown by animals that underwent FVB and to a lesser extent by mice from the RBB group (FVB vs HCO: p < 0.001, FVB vs ACO: p < 0.001, FVB vs TVB: p < 0.001, FVB vs RBB: p = 0.015). Moreover, the time spent with nest-building activities was significantly affected by the different treatments (χ^2^ = 9.726, df = 4, p = 0.045), yet, the effect was not strong enough to be significant in the *post hoc* analysis.

**Table 2:** Expression of natural behaviours in the home-cage analysed for 60 minutes directly following the respective treatments.

	Home-cage behaviour					Statistical analysis	
Parameter	HCO	ACO	TVB	RBB	FVB	KWH	p-value
feeding (%)	6.0 ± 1.3	11.5 ± 1.2	9.2 ± 1.6	7.9 ± 1.5	6.6 ± 1.9	χ^2^ = 7.126	0.129
grooming (%)	13.1 ± 1.7^a^	18.3 ± 1.6^a,b^	22.5 ± 2.3^a,b^	21.1 ± 1.7^a,b^	24.4 ± 4.3^b^	χ^2^ = 11.597	**0.021**
nest-building (%)	35.2 ± 6.1	36.9 ± 5.7	40.4 ± 4.7	32.4 ± 5.7	14.4 ± 6.1	χ^2^ = 9.739	**0.045**
resting (%)	13.3 ± 6.1	7.0 ± 3.1	2.9 ± 1.2	16.2 ± 6.1	8.2 ± 6.3	χ^2^ = 2.240	0.692
immobile with hunched posture (%)	0.0 ± 0.0^a^	0.0 ± 0.0^a^	0.0 ± 0.0^a^	0.0 ± 0.0^a^	27.8 ± 10.1^b^	χ^2^ = 26.061	**< 0.001**
locomotion (%)	16.0 ± 3.0	22.7 ± 4.7	18.2 ± 1.9	18.2 ± 3.1	17.0 ± 3.5	χ^2^ = 2.584	0.630
rearing (#)	46.1 ± 9.1^a,b^	51.5 ± 4.9^a^	48.6 ± 4.9^a,b^	30.9 ± 3.1^a,b^	26.0 ± 7.2^b^	χ^2^ = 14.601	**0.006**
miscellaneous (%)	11.3 ± 4.3^a^	2.1 ± 0.6^a,b^	4.7 ± 1.7^a,b^	3.3 ± 1.6^a,b^	0.7 ± 0.3^b^	χ^2^ = 10.641	**0.031**

Data are presented in percentage of time (over 60 minutes) except for rearing, which is presented as frequency (#). Data are depicted as means ± SEM**. Bold** typeface highlights significant differences between treatments (p < 0.05, df = 4; Kruskal-Wallis H test (KWH)). Different superscript letters denote significant differences between treatment groups (*post hoc* Bonferroni corrected Mann-Whitney U test, p < 0.05). HCO, handling control; ACO, anaesthesia control; TVB, tail vessel bleeding; RBB, retrobulbar bleeding; FVB, facial vein bleeding; SEM, standard error of the mean.

Interestingly, one day after the blood sampling, a significant difference was still found for nest-building behaviour (χ^2^ = 9.902, df = 4, p = 0.042). Here, the *post hoc* analysis revealed that mice that underwent FVB the previous day spent significantly less time with nest-building compared to mice from the RBB group (p = 0.037) (see S3 Table).

### The three blood sampling techniques did not affect body weight, coat state and food intake

Body weight, food intake and coat state were assessed as additional indicators of animal welfare. Overall, the body weight of the animals was not altered following blood sampling or control treatment in any of the experiments (experiment 2–4, see Table 3).

**Table 3:** Body weight of the mice in experiment 2–4.

		Body weight (g)					Statistical analysis	
Exp.	Time point	HCO	ACO	TVB	RBB	FVB	ANOVA	p-value
2	0 h	25.4 ± 0.6	25.7 ± 0.5	25.5 ± 0.4	26.2 ± 0.5	25.0 ± 0.5	F_(4,55)_ = 2.404	0.536
	24 h	25.5 ± 0.5	25.5 ± 0.5	25.3 ± 0.3	26.1 ± 0.5	24.7 ± 0.5	F_(4,55)_ = 1.124	0.355
3	-24 h	27.0 ± 0.2	26.8 ± 0.4	26.5 ± 0.3	26.8 ± 0.4	27.1 ± 0.2	F_(4,47)_ = 0.333	0.855
	0 h	27.1 ± 0.3	26.8 ± 0.4	26.6 ± 0.3	27.0 ± 0.4	27.1 ± 0.2	F_(4,47)_ = 0.369	0.829
	24 h	26.5 ± 0.3	26.6 ± 0.5	26.2 ± 0.3	26.5 ± 0.4	26.8 ± 0.3	F_(4,47)_ = 0.517	0.723
4	-24 h	26.5 ± 0.3	26.2 ± 0.5	26.7 ± 0.5	26.3 ± 0.5	26.1 ± 0.5	F_(4,44)_ = 0.206	0.934
	0 h	26.5 ± 0.4	26.2 ± 0.5	26.7 ± 0.5	26.2 ± 0.5	26.2 ± 0.5	F_(4,44)_ = 0.201	0.936
	24 h	26.4 ± 0.3	26.1 ± 0.5	26.5 ± 0.4	26.0 ± 0.4	25.9 ± 0.5	F_(4,44)_ = 0.189	0.943

Animals were weighed 24 hours before (-24 h, Exp. 3 & 4), immediately before (0 h, Exp. 2–4) and 24 hours after (24 h, Exp. 2–4) the respective treatment. Data are depicted as means ± SEM. No significant differences were detected between treatment groups (p > 0.05; Univariate ANOVA). ACO, anaesthesia control; FVB, facial vein bleeding; HCO, handling control; RBB, retrobulbar bleeding; TVB, tail vessel bleeding; SEM, standard error of the mean.

In addition, the coat state of the mice did not differ between the treatment groups immediately before and 24 hours after the respective treatment (see S4 Table). Similarly, food intake measured over 24 hours before and after the respective treatment did not differ significantly between the treatment groups (see S5 Table).

## Discussion

The present study aimed to investigate the effects of three commonly used blood sampling techniques in mice with respect to their impact on animal welfare. By applying a rigorous design in a comprehensive series of experiments, we could show that indeed, a single blood sampling with these three techniques affected the stress physiology and behaviour of the animals differentially and consistently elicited different degrees of distress, impacting parameters relevant for animal welfare assessment. These impacts seem to be stronger following FVB and RBB. Thus our results highlight TVB as more animal welfare friendly than the other two applied techniques.

While body weight, coat state, and food intake were not significantly different between the five treatment groups, a substantial corticosterone response was detected (Figs 2, 3 and 5). All groups showed an increase in plasma corticosterone levels compared to baseline. The reaction values 15 minutes after the respective treatment was lowest in the handling control and TVB groups. FVB and RBB showed the sharpest increase and also a slower recovery/prolonged activation, as demonstrated by the still increased levels of plasma corticosterone 75 minutes after the treatment (Fig 5). As plasma glucocorticoid levels are an indicator for the magnitude of the stress response [51], these results indicate that FVB and RBB are experienced as more stressful by laboratory mice than TVB or the control treatments. Even though the isoflurane anaesthesia itself elicited a stronger stress response than the handling, anaesthesia alone could not explain the substantial increase observed in animals that underwent RBB, as these two groups differ significantly from each other. These results are further supported by the analysis of faecal corticosterone metabolites (Fig 3). Corticosterone metabolites excreted eight—ten hours after exposure to a stimulus mirror the activity of the HPA axis in response to this stressor [44, 45]. Indeed, starting already six hours after the respective treatment, animals that underwent FVB had significantly higher FCM levels compared to HCO animals. After eight hours, this effect was even more pronounced: FVB and RBB treatment led to significantly higher levels compared to handling control, anaesthesia control, and TVB. After ten hours, RBB and FVB differed significantly from handling control. From these, it can be concluded that blood sampling from the *vena facialis* and retrobulbar venous sinus are significantly more stressful compared to TVB, HCO and ACO. These findings are in line with results reported by other groups [18, 24, 52, 53]. For example, Madetoja and colleagues could show that female mice which underwent tail vessel bleeding showed a weaker increase in plasma corticosterone levels compared to sampling from the *vena facialis* and the saphenous vein [52]. Similarly, other studies showed that unrestrained tail snips resulted in lower plasma corticosterone levels in comparison to anaesthetised tail snip and retro-orbital puncture [53] and plasma corticosterone concentrations were higher following RBB and sublingual puncture compared to tail tip amputation [18]. Other studies did not find these effects [17, 54, 55]. For example, Gjendal and colleagues investigated FCM concentrations following several blood sampling techniques, including RBB and FVB in female mice. Though they did not find significant increases, this is probably due to the fact that in their study samples were pooled over 24 hours following the treatment and not collected in shorter time intervals, i.e. teasing apart the time-course of the stress response [54]. In other studies, the discrepancies to our results are likely due to differences in blood sampling procedures, e.g., warming of the tail and restraining the animal for TVB [17]. However, when only small amounts of blood are needed, these extra measures are not necessary when applying TVB.

In line with the differences found in plasma corticosterone and FCM levels, animals that underwent FVB and to a lesser extend RBB, showed profound changes in their behaviour (in an experimental environment as well as in the home-cage). In general, following FVB, mice showed reduced locomotor activity and decreased interest in novel objects or social partners as demonstrated by the Open Field, Novel Object and Social Interaction tests (Fig 4, S1 and S2 Tables). Following RBB, a similar effect on the exploration of novel stimuli was found, and mice seemed to avoid the centre of the apparatus. However, overall locomotor activity was not reduced in animals of the RBB group. Interestingly, this altered behaviour was not observed anymore 24 hours later. These findings indicate that while FVB and RBB alter locomotor activity and exploration, these are acute effects and do not seem to have medium- or long-term implications.

The acute reduction in locomotor activity also fits to out observations in the home-cages of the animals, where the mice were left undisturbed, and their behaviour was recorded for one hour. While general locomotion did not differ between the treatment groups, it was a striking observation that some mice spent long times immobile with a hunched posture (i.e. stooped low with increased curvature of the back and their ears pulled back, the limbs pulled in under the body). This behaviour was almost exclusively exhibited by mice that experienced FVB and to a much lesser extent by mice that underwent RBB. This posture is viewed as an indicator for pain and distress in mice [56–58] and form a substantial impact on the animal’s welfare. In a study comparing blood collection by either tail vein incision, tail tip amputation or facial vein puncture it has also been shown, that in the 10 minutes following blood collection, FVB led to an increased frequency of inactive (i.e. at least 15 seconds of immobility or freezing) episodes [26]. Here, we showed that an increased inactivity is also observed in animals that underwent RBB.

Moreover, mice from the FVB group showed significantly more grooming in the home-cage than handling control mice, which might be due to the scruffing of the neck and the experience of pain in the craniofacial region that the FVB group experienced. When looking at the time course of nest-building activity over 24 hours, it becomes evident that FVB and RBB showed lower nest scores, i.e. reduced nest quality, four and six hours after the respective treatment. After that, nest scores no longer differed. A high degree of nest-building activity can be interpreted as a sign that all needs of the animals are met and conversely when nest-building is decreased as an indication for reduced welfare [20, 37, 39]. This is further evidence that FVB and RBB affect the welfare of mice as the nests they built during the first hours following blood sampling were of significantly poorer quality. In other studies, different blood sampling techniques, e.g., unrestrained tail snip, tail vessel bleeding, retrobulbar bleeding and facial vein bleeding did not lead to differences in nest-building behaviour. However, in these studies, nest quality was scored 10 hours [26] or 24 hours [54] after the blood sampling, which is in line with our findings, as we similarly saw no significant differences beyond 6 hours after the treatment. Also, the time to integrate nesting material into an already existing nest seems to be prolonged following a surgical intervention [59], further corroborating our findings. In contrast, another study could show that nest-building was similar between control and FVB treatment and significantly reduced after TVB and RBB [25]. One explanation for this discrepancy might be that in order to collect blood from the tail vessel, the mice in that study were physically restrained in a restrainer box. Although not fixated, this restraint might elicit a higher degree of distress than simply holding the otherwise freely moving mouse by the tail as done in our study. Moreover, Harikrishnan and colleagues used a needle for FVB and not a lancet [25]. However, as other studies did not find differences in behavioural measures after using a lancet or a needle [7], this explanation is speculative.

When looking at the scores of the MGS (Fig 7), it is evident as well that FVB and RBB seem to elicit the strongest stress response in the experimental mice. Initially, the MGS was developed to assess pain, but it also mirrors stress exposure [40, 60]. The MGS scores were highest in mice that experienced FVB and RBB, and these are the only treatments that differed significantly from the handling control group. However, it cannot be ruled out that the observed differences were caused by the manipulation in the facial region during FVB and RBB. Yet, the treatment was only performed on one side of the face while both sides were observed for the MGS scoring; therefore, we believe this potential effect to be minimal and to not have fully jeopardised the MGS measurements. Intriguingly, although not significant, animals that underwent TVB showed lower MGS scores compared to ACO mice. It seems that a brief exposure to anaesthesia alone has an effect on facial expression in C57BL/6J mice. These findings are in line with reports on strain-specific effects on MGS scores following isoflurane exposure [61]. Interestingly, other studies found more pronounced effects in female mice but not male mice following single and repeated isoflurane anaesthesia [60], yet single and repeated ketamine and xylazine anaesthesia produced significantly higher scores in male and female mice [62].

Taking the changes in behaviour together, a clear picture emerges that blood sampling from the facial vein and to a lesser degree from the retrobulbar sinus lead to welfare-relevant changes that are generally reflected in decreased activity of the mice. Such alterations in activity patterns in mice have been discussed as signs of distress [33, 63]. Similar to our findings, mice that underwent facial vein phlebotomy also showed a reduction in wheel-running behaviour, a behavioural activity recently suggested as a tool for severity assessment in laboratory mice [64].

Overall, it becomes apparent that sampling methods that are performed in the facial region (RBB, FVB) of the animal have more adverse effects on behaviour and stress physiology of mice. These results could be explained by the higher level of invasiveness of these methods, i.e. scruffing and anaesthesia in addition to the vein puncture. Another explanation might be the perception of pain. In humans, pain experienced in facial regions is usually rated as stronger compared to bodily pain and craniofacial pain is qualitatively different from extracranial nociception [65]. It was recently shown that this might also be the case in rodents [66]. Therefore, blood sampling in craniofacial regions in mice might elicit stronger affective pain than sampling from other parts of the body, e.g. the tail.

## Conclusion

Taken together, the data presented in our study provide ample and comprehensive evidence that the three commonly applied blood sampling techniques (TVB, RBB, FVB) affected the animals differently. Already a single blood sampling using FVB resulted in profound changes in physiological and behavioural stress parameters associated with reduced animal welfare. RBB led to similar results in the same direction. These effects were evident acutely after the respective treatments (i.e. in the first hours after blood sampling), but were no longer detectable 24 hours later, indicating only short-term impacts. Interestingly, TVB had the least impact on the animals and mostly did not even induce a significant deviation from the handling control group. Therefore, if only relatively small blood volumes (up to 150 μl) are needed and the quality of the blood sample (collection from the skin surface) does not interfere with the biochemical readouts, according to our results, TVB is the most animal welfare-friendly technique. Thus, by also taking the animal’s perspective and comprehensively assessing the severity of the particular sampling procedures, the results of our study provide a major contribution to *Refinement* within the 3R concept. This allows researchers to objectively select the most appropriate and welfare-friendly blood sampling technique for a given experiment.

## Supporting information

S1 Data Underlying data of presented results. Excel spreadsheet containing, in separate sheets, the underlying numerical data for Figs 2–7 and S1–S5 Tables.
(XLSX)

S1 Fig Novel object. Wire pencil holder (10 x 10 x 11 cm (L x W x H), DOKUMENT; Ikea, Germany) that is used as the novel object in the Novel Object Exploration test. During the Social Interaction test, the unfamiliar mouse is placed inside (Exp. 3 & 4).
(TIF)

S2 Fig Mouse grimace scale box. To investigate acute effects of the different treatments on pain grimace in Exp. 4, mice were placed in Mouse Grimace Scale boxes made from acrylic glass (10 x 10 x 5 cm (L x W x H) and the lids were closed. Mice were then video-recorded for 5 minutes.
(TIF)

S1 Table Immediate effects of the different blood sampling techniques or control treatments on behaviour in the Open Field (A), Novel Object (B) and Social Interaction test (C). Data are presented as means and SEM. **Bold** typeface indicates statistically significant differences between groups (p < 0.05; Univariate ANOVA). Means with different superscript letters differ significantly from each other (p < 0.05, Bonferroni *post hoc*).
(DOCX)

S2 Table Medium-term effects of the different blood sampling techniques or control treatments on behaviour in the Open Field (A), Novel Object (B) and Social Interaction test (C). Data are presented as means and SEM. **Bold** typeface indicates statistically significant differences between groups (p < 0.05; Univariate ANOVA). Means with different superscript letters differ significantly from each other (p < 0.05, Bonferroni *post hoc*).
(DOCX)

S3 Table Expression of spontaneous behaviour in the home-cage analysed for 60 minutes 24 hours before (A) and 24 hours after (B) the respective treatment. Data are presented in percentage of time (over 60 minutes) except rearing, which is presented as frequency (#). Data are depicted as means ± SEM. **Bold** typeface highlights significant differences between treatments (p < 0.05, df = 4; Kruskal-Wallis H test (KWH)). Means with different superscript letters differ significantly from each other (*post hoc* Bonferroni corrected Mann-Whitney U test, p < 0.05).
(DOCX)

S4 Table Coat state scores of the mice immediately before the respective treatment and 24 hours afterwards (score out of a maximum of seven). Depicted are mean ± SEM. No significant differences were detected between treatment groups (p > 0.05, df = 4 Kruskal-Wallis H test (KWH)).
(DOCX)

S5 Table Food intake over 24 hours before and after the respective treatment. Presented are means ± SEM. No significant differences were detected between treatment groups (p > 0.05, df = 4; Kruskal-Wallis H test (KWH)).
(DOCX)
