# Supplementary figures and images for: Impact of three commonly used blood sampling techniques on the welfare of laboratory mice: Taking the animal’s perspective
Source: PLoS One. 2020 Sep 8;15(9):e0238895. doi: 10.1371/journal.pone.0238895 (PMC7478650; doi:10.1371/journal.pone.0238895)

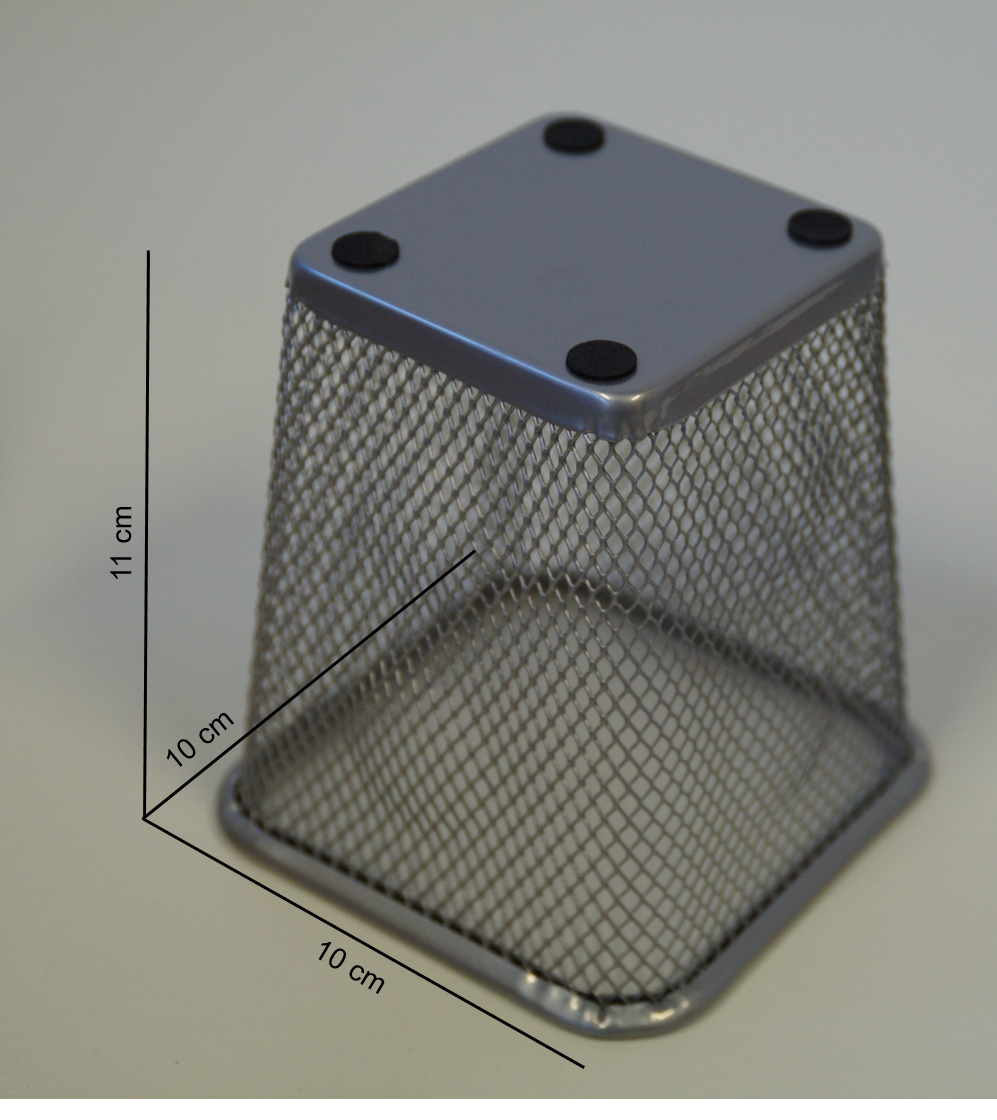

Supplement: S1 Fig — Wire pencil holder (10 x 10 x 11 cm (L x W x H), DOKUMENT; Ikea, Germany) that is used as the novel object in the Novel Object Exploration test. During the Social Interaction test, the unfamiliar mouse is placed inside (Exp. 3 & 4). (TIF) [file pone.0238895.s002.tif]

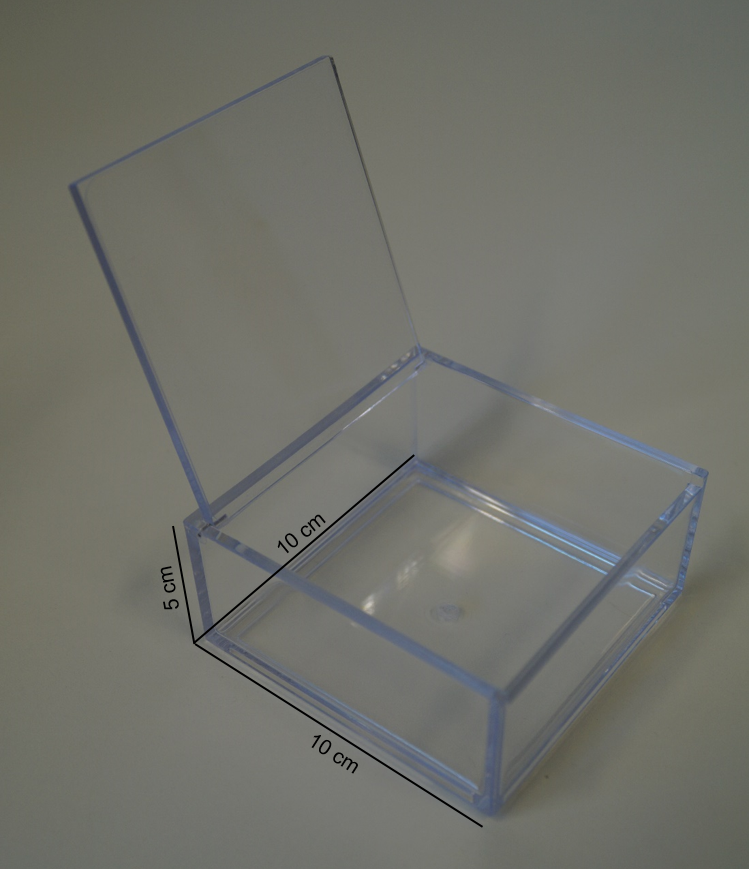

Supplement: S2 Fig — To investigate acute effects of the different treatments on pain grimace in Exp. 4, mice were placed in Mouse Grimace Scale boxes made from acrylic glass (10 x 10 x 5 cm (L x W x H) and the lids were closed. Mice were then video-recorded for 5 minutes. (TIF) [file pone.0238895.s003.tif]
