# Supplementary material for: Impact of three commonly used blood sampling techniques on the welfare of laboratory mice: Taking the animal’s perspective
Source: PLoS One. 2020 Sep 8;15(9):e0238895. doi: 10.1371/journal.pone.0238895 (PMC7478650; doi:10.1371/journal.pone.0238895)
Supplement: S1 Table — Immediate effects of the different blood sampling techniques or control treatments on behaviour in the Open Field (A), Novel Object (B) and Social Interaction test (C). Data are presented as means and SEM. Bold typeface indicates statistically significant differences between groups (p < 0.05; Univariate ANOVA). Means with different superscript letters differ significantly from each other (p < 0.05, Bonferroni post hoc). (DOCX) [file pone.0238895.s004.docx]

**S1 Table** **Immediate effects of different blood sampling techniques and control treatment on behaviour in the Open Field (A), Novel Object (B) and Social Interaction test (C)**. Data are presented as means ± SEM.  **Bold** typeface highlights statistically significant differences between groups (p < 0.05; Univariate ANOVA). Means with different letters differ significantly from each other (p < 0.05, Bonferroni *post hoc*).

A) Open Field test

|  |  | | | | | Statistical analysis | |
| --- | --- | --- | --- | --- | --- | --- | --- |
| Parameter | HCO | ACO | TVB | RBB | FVB | ANOVA | p-value |
| Total distance (m) | 28.6 ± 2.7^a^ | 27.0 ± 2.6 ^a^ | 25.8 ± 1.8^a^ | 28.6 ± 2.7^a^ | 12.5 ± 2.4^b^ | F_(4,46)_ = 7.830 | **< 0.001** |
| Entries to outer zone | 17.8 ± 2.2^a.b^ | 18.7 ± 2.0^a^ | 17.4 ± 2.5^a.b^ | 14.8 ± 2.3^a.b^ | 9.2 ± 2.1^b^ | F_(4,46)_ = 3.186 | **0.022** |
| Time in outer zone (s) | 261.5 ± 5.9 | 247.1 ± 12.8 | 262.9 ± 5.5 | 269.3 ± 5.5 | 273.0 ± 4.6 | F_(4,46)_ = 1.965 | 0.116 |
| Distance in outer zone (m) | 24.0 ± 2.7^a^ | 22.3 ± 2.4^a^ | 21.5 ± 2.0^a^ | 24.9 ± 2.5^a^ | 10.5 ± 2.0 | F_(4,46)_ = 6.641 | **< 0.001** |
| Entries to inner zone | 17.7 ± 2.3^a.b^ | 18.9 ± 1.9^a^ | 16.7 ± 2.4^a,b^ | 14.1 ± 2.4^a,b^ | 8.7 ± 2.2^b^ | F_(4,46)_ = 3.368 | **0.017** |
| Time in inner zone (s) | 34.1 ± 5.2 | 47.4 ± 12.3 | 33.6 ± 5.2 | 27.3 ± 5.0 | 24.0 ± 4.0 | F_(4,46)_ = 1.873 | 0.131 |
| Distance in inner zone (m) | 4.0 ± 0.6^a,b^ | 4.1 ± 0.5^a^ | 3.8 ± 0.6^a,b^ | 3.2 ± 0.7^a,b^ | 1.8 ± 0.5^b^ | F_(4,46)_ = 2.985 | **0.028** |
| Latency to enter inner zone (s) | 34.3 ± 11.5 | 34.7 ± 9.2 | 44.1 ± 13.3 | 34.2 ± 9.5 | 82.7 ± 20.4 | F_(4,46)_ = 1.706 | 0.165 |

B) Novel Object test

|  |  | | | | | Statistical analysis | |
| --- | --- | --- | --- | --- | --- | --- | --- |
| Parameter | HCO | ACO | TVB | RBB | FVB | ANOVA | p-value |
| Total distance (m) | 25.2 ± 2.1 | 24.8 ± 1.7^a^ | 20.6 ± 1.1 ^a^ | 21.4 ± 1.7^a^ | 13.5 ± 1.4^b^ | F_(4,46)_ = 8.669 | **< 0.001** |
| Entries to outer zone | 22.2 ± 1.9^a^ | 21.5 ± 2.4^a^ | 16.1 ± 1.8^a,b^ | 14.6 ± 2.4^a,b^ | 11.1 ± 1.9^b^ | F_(4,46)_ = 5.077 | **0.002** |
| Time in outer zone (s) | 152.7 ± 6.2^a^ | 165.1 ± 7.5^a^ | 183.7 ± 12.2^a,b^ | 220.6 ± 13.5^b^ | 221.7 ± 18.2^b^ | F_(4,46)_ = 6.375 | **< 0.001** |
| Distance in outer zone (m) | 12.7 ± 0.9^a.b^ | 13.3 ± 1.1^a,b^ | 12.3 ± 0.9^a,b^ | 14.9 ± 1.4^a^ | 9.1 ± 0.8^b^ | F_(4,46)_ = 4.498 | **0.004** |
| Entries to inner zone | 52.9 ± 5.2^a^ | 50.9 ± 4.0^a^ | 37.3 ± 4.5^a.b^ | 30.5 ± 5.7^b^ | 23.1 ± 5.6^b^ | F_(4,46)_ = 6.447 | **< 0.001** |
| Time in inner zone (s) | 42.7 ± 3.5 | 46.7 ± 5.2 | 39.5 ± 4.7 | 27.3 ± 5.4 | 28.7 ± 7.8 | F_(4,46)_ = 2.379 | 0.065 |
| Distance in inner zone (m) | 4.7 ± 0.7^a^ | 4.6 ± 0.5^a^ | 3.5 ± 0.5^a,b^ | 2.8 ± 0.6^a.b^ | 1.8 ± 0.5^b^ | F_(4,46)_ = 4.997 | **0.002** |
| Entries to interaction zone | 51.7 ± 4.9^a^ | 49.1 ± 2.4^a^ | 39.1 ± 4.1^a,b^ | 28.2 ± 4.5^b^ | 22.3 ± 5.2^b^ | F_(4,46)_ = 8.753 | **< 0.001** |
| Time in interaction zone (s) | 90.8 ± 6.5^a^ | 76.2 ± 8.8^a,c^ | 66.2 ± 7.1^a,b,c^ | 44.7 ± 8.0^b^ | 43.7 ± 9.8^b,c.^ | F_(4,46)_ = 6.200 | **< 0.001** |
| Latency to enter interact zone (s) | 20.9 ± 6.7^b^ | 29.5 ± 5.0^b,c^ | 33.6 ± 7.3^b,c^ | 93.4 ± 17.1^a^ | 82.5 ± 19.6^a,c^ | F_(4,46)_ = 6.619 | **< 0.001** |

C) Social Interaction test

|  |  | | | | | Statistical analysis | |
| --- | --- | --- | --- | --- | --- | --- | --- |
| Parameter | HCO | ACO | TVB | RBB | FVB | ANOVA | p-value |
| Total distance (m) | 16.3 ± 1.2 | 15.8 ± 1.3 | 14.5 ± 0.7 | 15.0 ± 1.5 | 11.8 ± 1.2 | F_(4,46)_ = 2.062 | 0.101 |
| Entries to outer zone | 12.9 ± 1.1 | 12.5 ± 1.2 | 10.1 ± 0.7 | 9.5 ± 1.0 | 9.1 ± 1.0 | F_(4,46)_ = 3.046 | **0.026** |
| Time in outer zone (s) | 127.1 ± 9.0^a^ | 127.7 ± 16.0^a^ | 155.1 ± 11.4^a,b^ | 198.5 ± 16.2^b^ | 182.0 ± 20.0^a,b^ | F_(4,46)_ = 4.399 | **0.004** |
| Distance in outer zone (m) | 8.2 ± 0.8 | 8.2 ± 1.1 | 9.0 ± 0.5 | 11.2 ± 1.6 | 7.6 ± 1.1 | F(_4,46)_ = 1.649 | 0.178 |
| Entries to inner zone | 33.6 ± 2.9^a^ | 29.0 ± 2.7^a,b^ | 23.1 ± 2.4^a.b^ | 17.9 ± 2.8^b^ | 18.7 ± 2.9^b^ | F_(4,46)_ = 6.058 | **0.001** |
| Time in inner zone (s) | 43.9 ± 4.4 | 42.4 ± 4.2 | 35.0 ± 5.1 | 27.2 ± 5.0 | 26.8 ± 3.8 | F_(4,46)_ = 3.286 | **0.019** |
| Distance in inner zone (m) | 2.5 ± 0.3^a^ | 2.4 ± 0.2^a^ | 1.8 ± 0.2^a,b^ | 1.3 ± 0.2^b^ | 1.3 ± 0.2^b^ | F_(4,46)_ = 5.966 | **0.001** |
| Entries to interaction zone | 38.9 ± 3.9^a^ | 39.7 ± 5.1^a^ | 30.1 ± 4.8^a,b^ | 20.7 ± 2.8^b^ | 25.8 ± 5.0^a,b^ | F_(4,46)_ = 4,230 | **0.005** |
| Time in interaction zone (s) | 123.1 ± 8.0^a^ | 119.3 ± 14.7^a,b^ | 106.0 ± 7.5^a,b^ | 73.0 ± 11.8^b^ | 84.1 ± 14.0^a,b^ | F_(4,46)_ = 3.513 | **0.014** |
| Latency to enter interact zone (s) | 23.9 ± 4.5^a^ | 22.6 ± 6.8^a^ | 49.3 ± 9.5^a,b^ | 75.5 ± 14.1^b^ | 55.4 ± 8.9^a,b^ | F_(4,46)_ = 5.622 | **0.001** |

Legend: HCO handling control, ACO, anaesthesia control, TVB tail vessel bleeding, RBB retrobulbar bleeding, FVB facial vein bleeding; m meter; s seconds; SEM standard error of the mean, interact. interaction
