# Supplementary material for: Impact of three commonly used blood sampling techniques on the welfare of laboratory mice: Taking the animal’s perspective
Source: PLoS One. 2020 Sep 8;15(9):e0238895. doi: 10.1371/journal.pone.0238895 (PMC7478650; doi:10.1371/journal.pone.0238895)
Supplement: S3 Table — Expression of spontaneous behaviour in the home-cage analysed for 60 minutes 24 hours before (A) and 24 hours after (B) the respective treatment. Data are presented in percentage of time (over 60 minutes) except rearing, which is presented as frequency (#). Data are depicted as means ± SEM. Bold typeface highlights significant differences between treatments (p < 0.05, df = 4; Kruskal-Wallis H test (KWH)). Means with different superscript letters differ significantly from each other (post hoc Bonferroni corrected Mann-Whitney U test, p < 0.05). (DOCX) [file pone.0238895.s006.docx]

**S3 Table** Expression of spontaneous behaviour in the home cage analysed for 60 minutes 24 hours before (A) and 24 hours after (B) the respective treatment. Data are presented in percentage of time (over 60 minutes) except rearing, which is presented as frequency (#). Data are depicted as means ± SEM**.** **Bold** typeface highlights significant differences between treatments (p < 0.05, df = 4, Kruskal-Wallis H test (KWH)). Means with different letters differ significantly from each other (*Post-hoc* Bonferroni corrected Mann-Whitney U test, p < 0.05).

A)

|  | Home cage behaviour 24 hours before treatment | | | | | Statistical analysis | |
| --- | --- | --- | --- | --- | --- | --- | --- |
| Parameter | HCO | ACO | TVB | RBB | FVB | KWH | p-value |
| feeding (%) | 7.9 ± 1.4 | 6,8 ± 1.2 | 9.5 ± 1.0 | 7.6 ± 1.3 | 9.5 ± 1.7 | χ^2^ = 3.389 | 0.495 |
| grooming (%) | 7.6 ± 1.4 | 7.9 ± 0.9 | 4.5 ± 0.7 | 7.1 ± 1.2 | 10.3 ± 1.3 | χ^2^ = 9.274 | 0.055 |
| nest-building (%) | 53.6 ± 6.0 | 61.0 ± 2.1 | 59.6 ± 5.0 | 60.3 ± 3.1 | 52.9 ± 3.0 | χ^2^ = 4.305 | 0.366 |
| resting (%) | 5.5 ± 3.3 | 2.5 ± 2.1 | 0.9 ± 0.6 | 1.9 ± 0.9 | 1.2 ± 0.6 | χ^2^ = 0.988 | 0.912 |
| inactivity with hunched posture (%) | 0.0 ± 0.0 | 0.0 ± 0.0 | 0.0 ± 0.0 | 0.0 ± 0.0 | 0.0 ± 0.0 | χ^2^ < 0.001 | > 0.999 |
| locomotion (%) | 16.6 ± 2.5 | 17.0 ± 2.1 | 20.5 ± 4.7 | 15.7 ± 1.9 | 18.7 ± 0.9 | χ^2^ = 1.512 | 0.825 |
| rearing (#) | 41.0 ± 5.1 | 42.1 ± 3.4 | 40.2 ± 3.4 | 32.1 ± 3.5 | 42.9 ± 3.2 | χ^2^ = 5.469 | 0.242 |
| miscellaneous (%) | 7.5 ± 2.7 | 3.6 ± 1.1 | 3.4 ± 0.8 | 6.3 ± 1.5 | 2.6 ± 0.3 | χ^2^ = 6,467 | 0.167 |

B)

|  | Home cage behaviour 24 hours after treatment | | | | | Statistical analysis | |
| --- | --- | --- | --- | --- | --- | --- | --- |
| Parameter | HCO | ACO | TVB | RBB | FVB | KWH | p-value |
| feeding (%) | 9.2 ± 1.2 | 6.6 ± 1.9 | 7.5 ± 1.6 | 8.6 ± 1.9 | 11.8 ± 2.8 | χ^2^ = 2.770 | 0.597 |
| grooming (%) | 13.2 ± 2.8 | 14.7 ± 1.9 | 17.1 ± 2.5 | 12.5 ± 1.4 | 16.3 ± 1.9 | χ^2^ = 4.474 | 0.346 |
| nest-building (%) | 31.9 ± 4.7^a,b^ | 40.4 ± 3.8^a,b^ | 34.6 ± 5.3^a,b^ | 43.1 ± 4.6^a^ | 21.9 ± 4.8^b^ | χ^2^ = 9.902 | **0.042** |
| resting (%) | 18.4 ± 5.8 | 2.6 ± 0.8 | 9.8 ± 4.7 | 7.0 ± 4.5 | 20.5 ± 4.5 | χ^2^ = 7.689 | 0.104 |
| inactivity with hunched posture (%) | 0.0 ± 0.0 | 0.0 ± 0.0 | 0.0 ± 0.0 | 0.0 ± 0.0 | 0.0 ± 0.0 | χ^2^ < 0.001 | > 0.999 |
| locomotion (%) | 20.6 ± 3.2 | 25.8 ± 3.3 | 22.3 ± 3.5 | 18.7 ± 2.6 | 24.5 ± 3.2 | χ^2^ = 2.359 | 0.670 |
| rearing (#) | 47.7 ± 5.9 | 59.3 ± 7.8 | 55.3 ± 7.1 | 57.3 ± 9.9 | 48.4 ± 5.7 | χ^2^ = 2.013 | 0.733 |
| miscellaneous (%) | 5.4 ± 2.9 | 8.2 ± 1.9 | 7.5 ± 2.5 | 9.1 ± 3.2 | 4.0 ± 0.9 | χ^2^ = 3.368 | 0.498 |

Legend: HCO handling control, ACO, anaesthesia control, TVB tail vessel bleeding, RBB retrobulbar bleeding, FVB facial vein bleeding; SEM standard error of the mean
