# Supplementary material for: Impact of three commonly used blood sampling techniques on the welfare of laboratory mice: Taking the animal’s perspective
Source: PLoS One. 2020 Sep 8;15(9):e0238895. doi: 10.1371/journal.pone.0238895 (PMC7478650; doi:10.1371/journal.pone.0238895)
Supplement: S4 Table — Depicted are mean ± SEM. No significant differences were detected between treatment groups (p > 0.05, df = 4 Kruskal-Wallis H test (KWH)). (DOCX) [file pone.0238895.s007.docx]

**S4 Table** Coat state scores of the mice immediately before the respective treatment and 24 hours afterwards (score out of a maximum of seven). Depicted are mean ± SEM. No significant differences were detected between treatment groups (p > 0.05, df = 4, Kruskal-Wallis H test (KWH)).

|  | Coat state score | | | | | Statistical analysis | |
| --- | --- | --- | --- | --- | --- | --- | --- |
|  | HCO | ACO | TVB | RBB | FVB | KWH | p-value |
| before treatment | 0.0 ± 0.0 | 0.0 ± 0.0 | 0.1 ± 0.1 | 0.1 ± 0.1 | 0.0 ± 0.0 | χ^2^ = 3.108 | 0.540 |
| 24 h after treatment | 0.0 ± 0.0 | 0.1 ± 0.1 | 0.1 ± 0.1 | 0.3 ± 0.1 | 0.1 ± 0.1 | χ^2^ = 4.822 | 0.306 |

Legend: HCO, handling control; ACO, anaesthesia control; TVB, tail vessel bleeding; RBB, retrobulbar bleeding; FVB, facial vein bleeding; SEM, standard error of the mean
