# Supplementary material for: Impact of three commonly used blood sampling techniques on the welfare of laboratory mice: Taking the animal’s perspective
Source: PLoS One. 2020 Sep 8;15(9):e0238895. doi: 10.1371/journal.pone.0238895 (PMC7478650; doi:10.1371/journal.pone.0238895)
Supplement: S5 Table — Presented are means ± SEM. No significant differences were detected between treatment groups (p > 0.05, df = 4; Kruskal-Wallis H test (KWH)). (DOCX) [file pone.0238895.s008.docx]

**S5 Table** Food intake over the 24 hour before and after the respective treatment. Depicted are mean ± SEM. No significant differences were depicted between treatment groups (p > 0.05, df = 4, Kruskal-Wallis H test (KWH)).

|  | Food intake (g) | | | | | Statistical analysis | |
| --- | --- | --- | --- | --- | --- | --- | --- |
|  | HCO | ACO | TVB | RBB | FVB | KWH | p-value |
| 24 h before treatment | 6.1 ± 0.2 | 6.0 ± 0.2 | 5.9 ± 0.2 | 5.6 ± 0.2 | 5.7 ± 0.2 | χ^2^ = 3.853 | 0.426 |
| 24 h after treatment | 5.5 ± 0.5 | 5.2 ± 0.2 | 5.1 ± 0.2 | 4.6 ± 0.6 | 5.0 ± 0.2 | χ^2^ = 2.479 | 0.648 |

Legend: ACO, anaesthesia control; df, degrees of freedom; FVB, facial vein bleeding; HCO, handling control; RBB, retrobulbar bleeding; SEM, standard error of the mean; TVB, tail vessel bleeding
